# Micro-Particle Image Velocimetry Investigation of Flow Fields of SonoVue Microbubbles Mediated by Ultrasound and Their Relationship With Delivery

**DOI:** 10.3389/fphar.2019.01651

**Published:** 2020-01-28

**Authors:** Penglin Zou, Mengqi Li, Ziqi Wang, Guoxiu Zhang, Lifang Jin, Yan Pang, Lianfang Du, Yourong Duan, Zhaomiao Liu, Qiusheng Shi

**Affiliations:** ^1^Department of Ultrasound, Shanghai General Hospital, Shanghai Jiao Tong University School of Medicine, Shanghai, China; ^2^College of Mechanical Engineering and Applied Electronics Technology, Beijing University of Technology, Beijing, China; ^3^Department of Emergency, The First Affiliated Hospital of Henan University of Science and Technology, Luoyang, China; ^4^State Key Laboratory of Oncogenes and Related Genes, Shanghai Cancer Institute, Renji Hospital, Shanghai Jiao Tong University School of Medicine, Shanghai, China

**Keywords:** flow field, microbubble, delivery, ultrasound, shear stress

## Abstract

The flow fields generated by the acoustic behavior of microbubbles can significantly increase cell permeability. This facilitates the cellular uptake of external molecules in a process known as ultrasound-mediated drug delivery. To promote its clinical translation, this study investigated the relationships among the ultrasound parameters, acoustic behavior of microbubbles, flow fields, and delivery results. SonoVue microbubbles were activated by 1 MHz pulsed ultrasound with 100 Hz pulse repetition frequency, 1:5 duty cycle, and 0.20/0.35/0.70 MPa peak rarefactional pressure. Micro-particle image velocimetry was used to detect the microbubble behavior and the resulting flow fields. Then HeLa human cervical cancer cells were treated with the same conditions for 2, 4, 10, 30, and 60 s, respectively. Fluorescein isothiocyanate and propidium iodide were used to quantitate the rates of sonoporated cells with a flow cytometer. The results indicate that (1) microbubbles exhibited different behavior in ultrasound fields of different peak rarefactional pressures. At peak rarefactional pressures of 0.20 and 0.35 MPa, the dispersed microbubbles clumped together into clusters, and the clusters showed no apparent movement. At a peak rarefactional pressure of 0.70 MPa, the microbubbles were partially broken, and the remainders underwent clustering and coalescence to form bubble clusters that exhibited translational oscillation. (2) The flow fields were unsteady before the unification of the microbubbles. After that, the flow fields showed a clear pattern. (3)The delivery efficiency improved with the shear stress of the flow fields increased. Before the formation of the microbubble/bubble cluster, the maximum shear stresses of the 0.20, 0.35, and 0.70 MPa groups were 56.0, 87.5 and 406.4 mPa, respectively, and the rates of the reversibly sonoporated cells were 2.4% ± 0.4%, 5.5% ± 1.3%, and 16.6% ± 0.2%. After the cluster formation, the maximum shear stresses of the three groups were 9.1, 8.7, and 71.7 mPa, respectively. The former two could not mediate sonoporation, whereas the last one could. These findings demonstrate the critical role of flow fields in ultrasound-mediated drug delivery and contribute to its clinical applications.

## Introduction

A microbubble population driven by ultrasound can enhance the delivery of external molecules through a process called ultrasound-mediated drug delivery (UMDD) (Tang et al., 2017). Numerous preclinical studies have proven the feasibility of UMDD and its advantages of safety, efficiency, and convenience (Shi et al., 2014; Xing et al., 2016; Lin et al., 2018). Through further research and development, UMDD has entered the clinical trial stage. Good therapeutic effect and no significant adverse effects have been observed (Carpentier et al., 2016; Dimcevski et al., 2016), which indicates that UMDD has great clinical potential. However, many obstacles still need to be overcome before clinical application can be achieved. One of the most important obstacles is that the exact mechanism has not been elucidated (Helfield et al., 2016b).

Cavitation, the creation and subsequent dynamic behavior of microbubbles, has been shown to play a key role in UMDD (De Cock et al., 2016; Huang et al., 2018; Pereno et al., 2018). According to the morphological changes of microbubbles, cavitation can be divided into two types: stable and transient. Stable cavitation refers to the periodic expansion and contraction of microbubbles around their equilibrium radius in a low-pressure sound field. Transient cavitation refers to the large expansion and rapid collapse of microbubbles in a high-pressure sound field (Huang et al., 2018). Because microbubbles are located within a medium or blood, the above behavior inevitably disturbs the surrounding liquid to form flow fields. The flow fields exert shear and normal stresses on the plasma membranes and induce pore formation (Rong et al., 2018). This process called sonoporation is the primary mechanism of UMDD (Mehier-Humbert et al., 2005; Kooiman et al., 2014; Qin et al., 2018). Depending on the duration of existence of the pores, sonoporation is divided into two types: reversible and irreversible (Wu et al., 2002; Lentacker et al., 2014; Nejad et al., 2016; Qin et al., 2018). The former refers to the formation of reparable pores on cell membranes, while the latter refers to irreparable pores. Both pore types can facilitate the cellular uptake of external impermeable macromolecules, but the former does not have a significant effect on cell viability, while the latter leads to cell death. Current research suggests that the duration of pores which varies from the order of milliseconds to minutes (Deng et al., 2004; Qin et al., 2016) depends on the pore size. The pore size varies from the order of nanometers to micrometers (Mehier-Humbert et al., 2005; Yun et al., 2009) and depends on the bubble-cell distance and peak negative pressure (PRP). For details of the spatiotemporal characteristics of pores, see Refs. (Qin et al., 2018). The acoustic behavior of microbubbles and the cell responses during the delivery process have been extensively studied, but the role of flow fields in delivery is largely unknown (Kooiman et al., 2014).

There have been few experimental studies on the flow fields of ultrasound-activated microbubbles, which may be due to the difficulty of quantitative analysis of microscale flow fields with sufficient spatial and temporal resolution. Optical microscopy can only observe the dynamic behavior of individual microbubbles and microbubble groups (Fan et al., 2014; Nejad et al., 2016; van Rooij et al., 2016) but cannot detect the velocity fields around them. Gelderblom (2012) used ultra-high-speed fluorescence imaging to capture acoustic streaming around liposome-loaded microbubbles, but this was only a qualitative study, and they did not provide detailed information. Marmottant and Hilgenfeldt (2003) added lipid vesicles as a tracer to a cuvette with oscillating bubbles attached and depicted the streamlines by tracking the trajectories of the vesicles. However, the size of the vesicles was 10–100 μm, which resulted in poor followability and significant interference to the flow fields. Thus, there was a large difference between the measured and calculated values of the flow fields. None of the above methods can meet the requirements of microscale flow field detection. Thus, flow visualization technology is required for in-depth research.

Micro-particle image velocimetry (Micro-PIV) is a microscale flow measurement technique developed in the 1990s (Raffel et al., 2007). It combines traditional PIV technology with optical microscopy and can accurately measure two-dimensional microscale velocity fields without interference. Currently, other measurement techniques such as micro-laser Doppler velocimetry, Raman scattering, and molecular tagging velocimetry do not provide a resolution and measurement accuracy comparable to micro-PIV. Tho et al. (2007) and Collis et al. (2010) used micro-PIV to study the flow fields around a bubble undergoing stable cavitation; they found that many different microstreaming patterns were possible around a bubble, and each pattern generated different shear stress and stretch/compression distributions. Reuter et al. (2017) used micro-PIV and high-speed cameras to study the flow fields and vortex dynamics of bubbles collapsing near a solid boundary. Their results showed that the flow patterns of transient cavitation included free and wall vortices and depended on the bubble stand-off distance. These experiments demonstrated the feasibility and accuracy of micro-PIV, but the research objects were individual unencapsulated bubbles of several hundred microns in diameter. In contrast, UMDD uses encapsulated microbubbles with a diameter of 10 µm or less. Cho et al. (2015) measured the movement speed of SonoVue microbubbles caused by primary and secondary radiation forces in a blood vessel model, but they ignored the flow fields generated by the oscillation of microbubbles and microbubble clusters. Pereno et al. (2018) developed an device called a layered acoustofluidic resonator. This device could optically and acoustically characterize cavitation dynamics, microstreaming, and biological effects simultaneously and was therefore an ideal system to study the interactions between UMDD and tissue. However, only the feasibility of the device was verified. The relationships among the detected objects were not studied, and no follow-up studies have been reported yet. Therefore, further research is needed on the flow fields of ultrasound-activated microbubbles.

In this study, bright field and fluorescence imaging were used on the micro-PIV system to capture the acoustic behavior and flow fields of SonoVue microbubbles driven by ultrasound, respectively. The relationships among the ultrasound parameters, acoustic behavior of microbubbles, and flow fields were clarified through qualitative and quantitative analyses of the flow patterns and shear microenvironment. Fluorescein isothiocyanate (FITC) was then delivered to HeLa human cervical cancer cells under the same experimental conditions, and the uptake efficiency and cell viability were analyzed with a flow cytometer. Based on the micro-PIV results and flow cytometry data, the role of the flow fields in UMDD was discussed. This study explored the mechanism of UMDD from the perspective of fluid dynamics, which not only contributes to the optimization, design, and future clinical transformation of this technology but also breaks a new path for research on its mechanism.

## Materials and Methods

### Ultrasound Exposure Device

**Figure 1:**
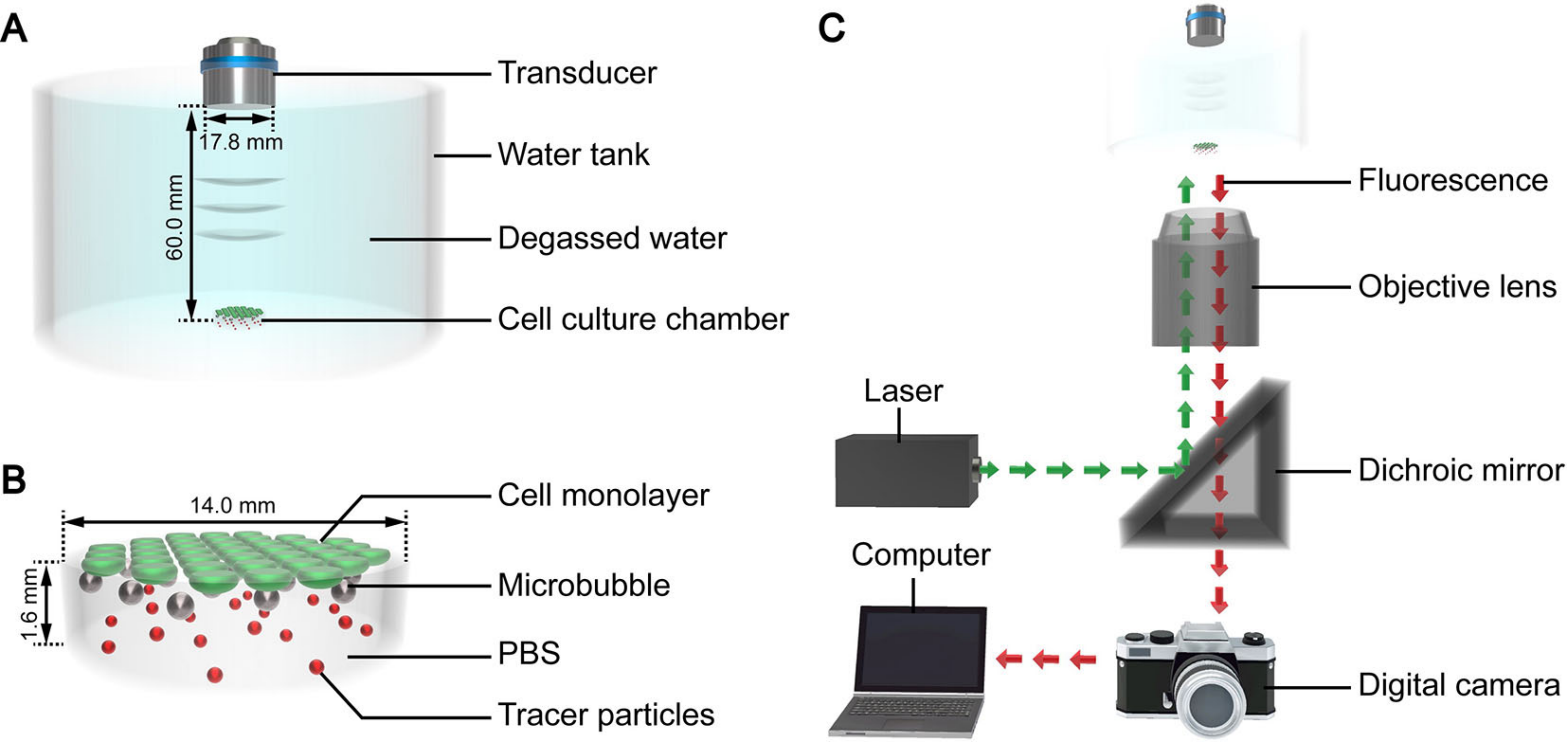
3D schematic diagram of the **(A)** ultrasound exposure device, **(B)** cell culture chamber, and **(C)** micro-PIV system.

The authors developed a custom device to facilitate ultrasound exposure of the SonoVue microbubbles, as shown in **Figure 1A**. Polydimethylsiloxane was used to fabricate a tank filled with degassed water for coupling the ultrasound. A cylindrical cell culture chamber (**Figure 1B**) made of borosilicate glass was placed at the bottom of the tank. The thickness of the glass was 0.13 mm, and the basal diameter and height of the chamber were 14 mm and 1.6 mm, respectively. A flat transmitting transducer (Physioson-Basic, PHYSIOMED Elektro-medizin, Germany) was vertically immersed in the degassed water using a three-dimensional fixator. The transducer had a diameter of 17.8 mm and completely covered the chamber. The distance of the acoustic near field in this experiment was about 54.9 mm. The sound pressure in the near field fluctuated greatly. Therefore, the chamber was placed in the far field at a distance of 60 mm from the incident surface of the transducer.

### Acoustic Calibration

PRP is one of the main influencing factors of sonoporation (Kotopoulis and Postema, 2010; Lin et al., 2017; Qin et al., 2018), so it was chosen as the research variable. Before the experiment proper commenced, the PRP was determined with a hydrophone (HGL-0200, ONDA, USA) at different output intensities. All experiments were carried out at a room temperature of 25 °C unless noted otherwise. Please see the **Supplementary Material** for details of the calibration.

### Microbubble Preparation

SonoVue microbubbles (Bracco, Italy) consist of phospholipid shells filled with sulfur hexafluoride and have a diameter of 2–7 µm. According to the manufacturer's specifications, the microbubbles were freshly reconstituted in 5 mL of physiological saline solution to form a suspension with a concentration of 2–5 × 10^8^/mL (Correas et al., 2001). The reconstituted SonoVue microbubbles were diluted in phosphate buffer saline (PBS) to a concentration of 20% (v/v).

### Experimental Grouping

The control groups for the studies consisted of the PBS group, US group, and MB group. The fluids to be tested of the three groups were PBS, PBS, and diluted microbubble suspension, respectively. And only the US group was exposed to ultrasound at a PRP of 0.70 MPa. The experimental groups had the same test fluid as the MB group, with ultrasound irradiation at PRPs of 0.20, 0.35, and 0.70 MPa. The PRP for UMDD typically ranges from 0.06 to 0.60 MPa (Fan et al., 2014; Qin et al., 2016; Pereno et al., 2018), with early *in vitro* studies reporting sonoporation at PRPs as high as 1.32 MPa (Lai et al., 2006). Within this range, we selected a number of PRP levels for pre-experiments. The results showed that 0.20 MPa was below the transient cavitation threshold and shear stress threshold for sonoporation, 0.35 MPa was below the transient cavitation threshold and above the shear stress threshold for sonoporation, 0.70 MPa was above the transient cavitation threshold and shear stress threshold for sonoporation. Therefore, we selected the above three levels with typical characteristics for formal experiments and reporting. The other ultrasound parameters were fixed to optimized values that were more suitable for delivery (Chen et al., 2016; Lin et al., 2018), including a center frequency of 1 MHz, pulse repetition frequency of 100 Hz, and duty cycle of 1:5.

### Micro-PIV Detection

The micro-PIV system (Dantec Dynamics, Denmark) consisted of an inverted microscope, double-pulse Nd : YAG laser, metal halide lamp, digital charge-coupled device camera, and computer (**Figure 1B**). The laser produced a laser beam with a wavelength of 532 nm. The laser beam was reflected by a dichroic mirror and then focused by the objective lens into the fluid to be tested in the cell culture chamber on the stage. After being excited by the laser beam, the tracer particles dispersed in the fluid emitted fluorescence at a wavelength of 612 nm, which was recorded by the camera. The computer processed the fluorescence and calculated the fluid velocity of the detected plane. The optical path of the metal halide lamp was similar to that of the laser and was mainly used for the bright field imaging of the acoustic behavior of the microbubble groups.

Monolayer cells are only few microns thick, which cannot significantly affect the acoustic interaction (Brujan et al., 2001). Therefore, cells were not seeded in the chamber in this section to ensure the accuracy of the micro-PIV detection. 0.02 mL of tracer particle suspension (Fluoro-Max, Thermo Fisher Scientific, USA) was added to each milliliter of the fluid to be tested. The ultrasound exposure device was placed on the stage of the micro-PIV system. Then, the mixed suspension was added into the chamber and allowed to stand for 100 s to ensure that all microbubbles floated to the top of the chamber. Subsequently, the vertical height of the objective lens was adjusted to focus on the plane of the microbubbles, which was the detection plane. The metal halide lamp and ultrasound were then simultaneously turned on to capture the bright field images of the microbubbles. Finally, the new mixed suspension was replaced, and the fluorescence of the detection plane was induced with laser irradiation.

The tracer particles used for flow visualization were 1 µm diameter polystyrene fluorescent microspheres that were coated in a red dye with an excitation wavelength of 532 nm. The size and dosage of the tracer particles were determined based on our previous researches (Liu et al., 2017; Liu et al., 2019) and pre-experiments in order to ensure the measurement accuracy. The numerical aperture of the objective lens was 0.4, and the magnification was 10 ×. The depth of correlation is given by $\delta_{DOC}=2{\left\{\frac{\left(1-\sqrt{\epsilon }\right)}{\sqrt{\epsilon }}\left[\frac{n_{o}^{2}d_{p}^{2}}{4d}+\frac{5.95{\left(M+1\right)}^{2}\lambda^{2}n_{o}^{4}}{16M^{2}d^{4}}\right]\right\}}^{1/2}$,where *d*_*p*_ is the particle diameter, λ is the laser wavelength, *M* is the magnification, *n*_*o*_ is the refractive index of the infiltrating liquid of the objective lens, *d* is the numerical aperture, and *ϵ* is the weight limit value (Olsen and Adrian, 2000). Thus, the depth of correlation in this experiment was 18 μm. The digital camera was used to acquire the bright and flow field images in single and dual frame modes, respectively, at a frequency of 6.1 Hz. The time interval between the two images in the dual frame mode was set according to different flow conditions from 50 to 3,000 µs (Liu et al., 2017; Liu et al., 2019). The fluorescence signal was analyzed with a standard cross-correlation algorithm implemented in commercial PIV software. The interrogation area size was 32 × 32 pixels with an overlap of 50%.

### Shear Stress Calculation

The velocity gradients were calculated from the measured velocities with the least squares method. Based on the velocity gradients, the shear rates were estimated as $\epsilon =\frac{\partial \overset{¯}{u}}{\partial y}+\frac{\partial \overset{¯}{v}}{\partial x}$ (Ma et al., 2015). The shear stress *τ* was calculated as *τ* = *ηϵ*, where *η* was the fluid viscosity and set to 1.05 ± 0.01 mPa·s (Helfield et al., 2016a).

### UMDD Delivery

HeLa cells are commonly used for UMDD (Lai et al., 2006; Qin et al., 2014; Fan et al., 2017) because they have a fast proliferation rate and are easy to operate experimentally. Therefore, these cells were selected in this study for the delivery experiments. HeLa cells were obtained from the Institute of Biochemistry and Cell Biology of the Chinese Academy of Sciences (Shanghai, China). Cells were seeded in the cell culture chamber and cultured in Dulbecco's modified Eagle medium (DMEM; Hyclone, USA) supplemented with 10% fetal bovine serum (Gibco, USA) in a humidified incubator at 37°C with 5% CO_2_. After the cells reached 60–70% confluence, the DMEM was removed. Then, a mixture of FITC (Invitrogen, USA) and the fluid to be tested was added. The final concentration of FITC was 2 mg/mL. After standing for 100 s, each sample was subjected to ultrasound irradiation. The irradiation duration was 60 s for the US group and 2, 4, 10, 30, or 60 s for the UMDD groups. After exposure to ultrasound, samples were placed in an incubator for 30 min.

### Evaluation of the FITC Uptake Efficiency and Cell Viability

To assess the cell viability after UMDD treatments, propidium iodide (PI; Invitrogen, USA) was added to the chamber at a concentration of 2 µM to stain the dead cells for 10 min. After being washed twice with PBS, the cells were trypsinized and collected for measurement of the FITC uptake efficiency and cell viability *via* fluorescence activated cell-sorting (Beckman Coulter, Miami, Florida).

### Statistical Analysis

All experiments were performed in triplicate. Statistical analysis of the fluorescence activated cell-sorting data was performed through one-way analysis of variance followed by Bonferroni multiple comparison with SPSS version 22.0. The significance level was set to a value of 0.05.

## Results and Discussion

### Relationships Between the Ultrasound Parameters and Acoustic Behavior of Microbubbles

**Figure 2:**
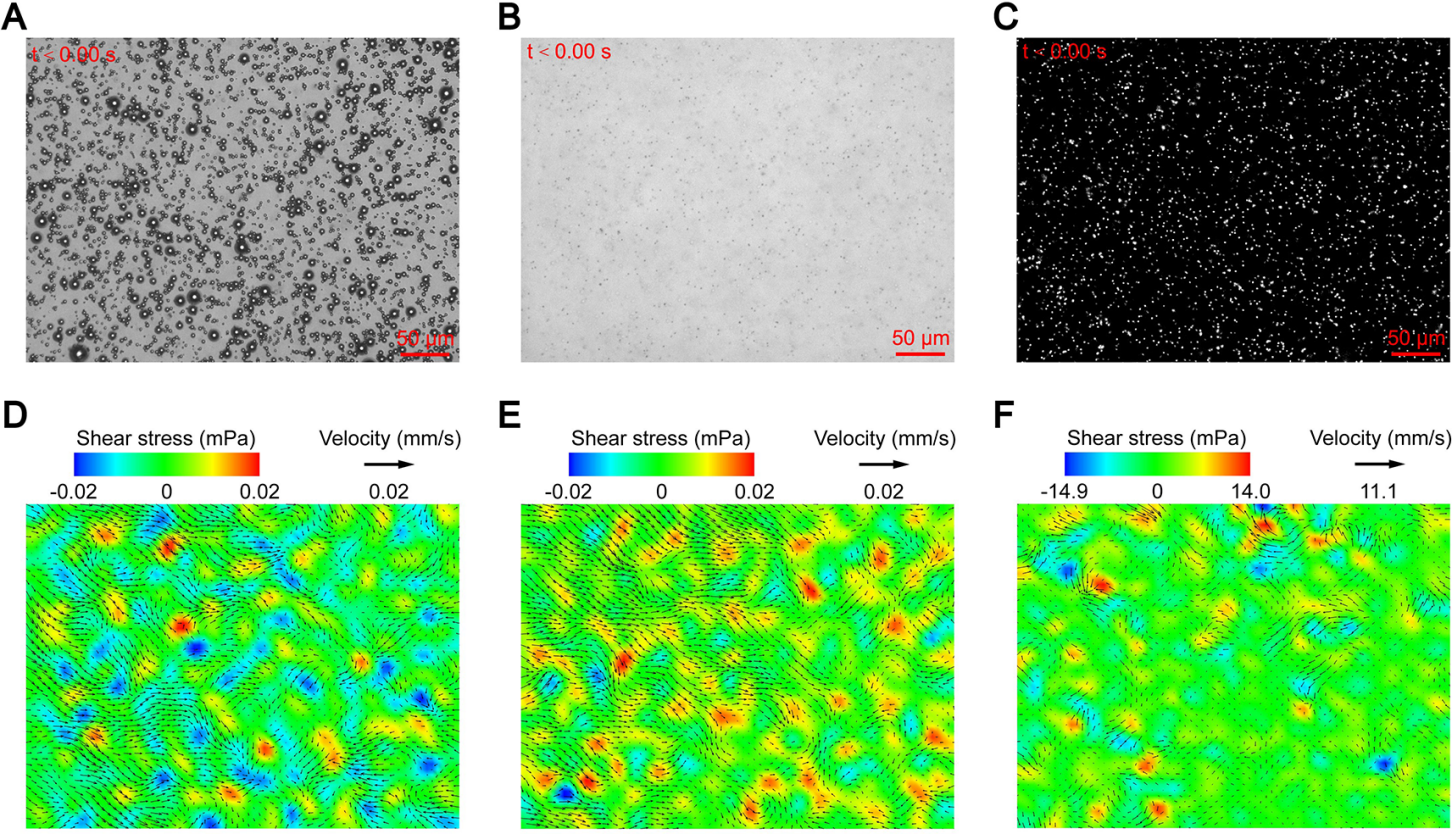
Micro-PIV results of the control groups: **(A)** suspended microbubbles at the detection plane; **(B)** flow tracers dispersed throughout the fluid; **(C)** fluorescent image of flow tracers at the detection plane; and maximum flow velocity and corresponding shear stress of the **(D)** PBS group, **(E)** MB group, and **(F)** US group.

After the suspension was added to the chamber and allowed to stand for 100 s, bright field imaging showed that the microbubbles of each group were uniformly and stably suspended at the detection plane (**Figure 2A**). The flow tracers were evenly dispersed throughout the fluid, and no significant floating or sinking occurred (**Figure 2B**). This was because that the particles matched the density of the surrounding fluid, which could minimize the measurement error caused by gravity and buoyancy (Tho et al., 2007).

**Figure 3:**
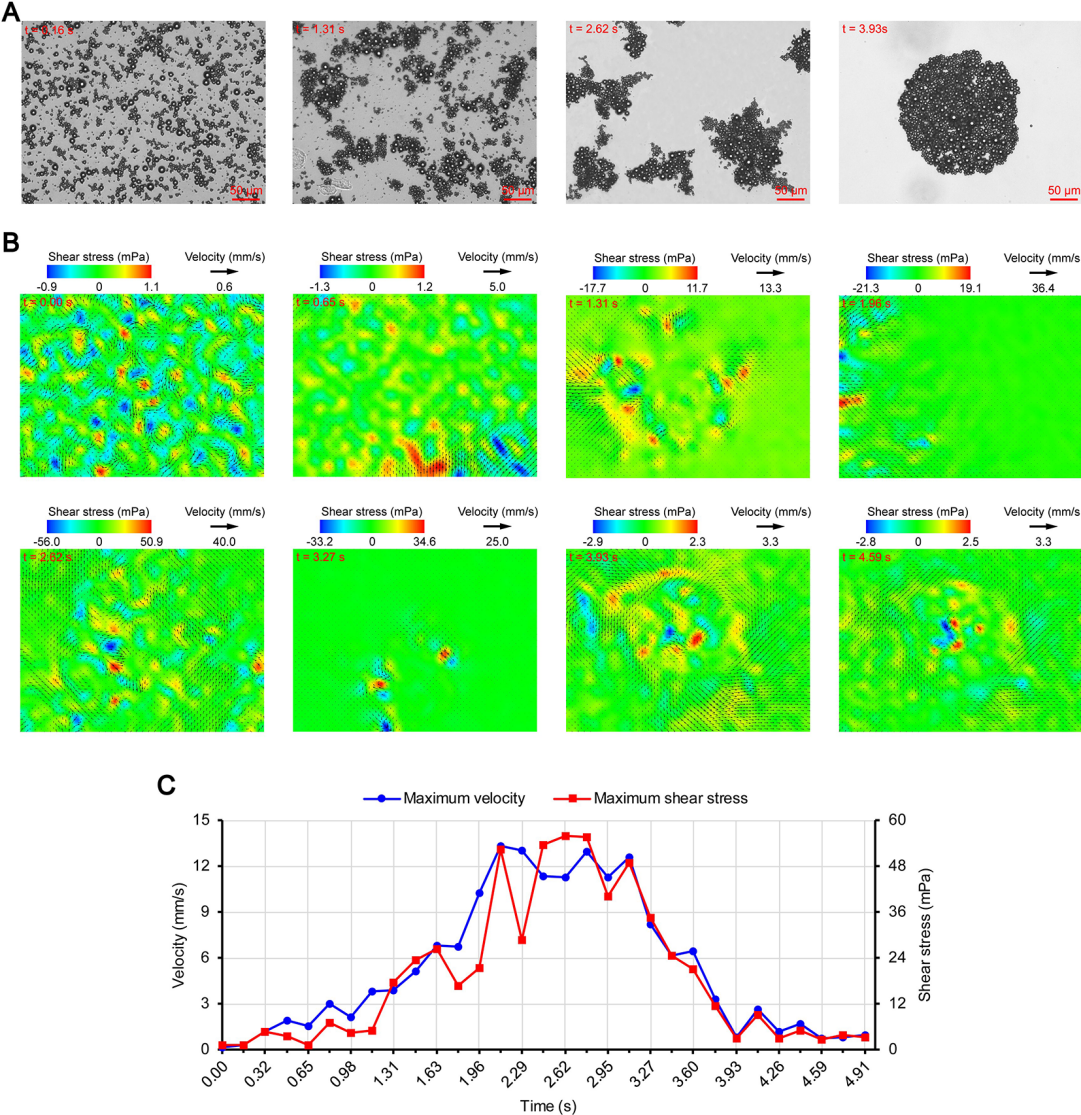
Micro-PIV results of the 0.20 MPa group. **(A)** Acoustic behavior of the microbubbles. The effect of the microbubbles on light was attenuated with accumulation, and the brightness of the images gradually increased. **(B)** Temporal evolution and spatial distribution of the velocity fields and shear stress. **(C)** Time variation of the maximum shear stress and maximum flow velocity.

**Figure 4:**
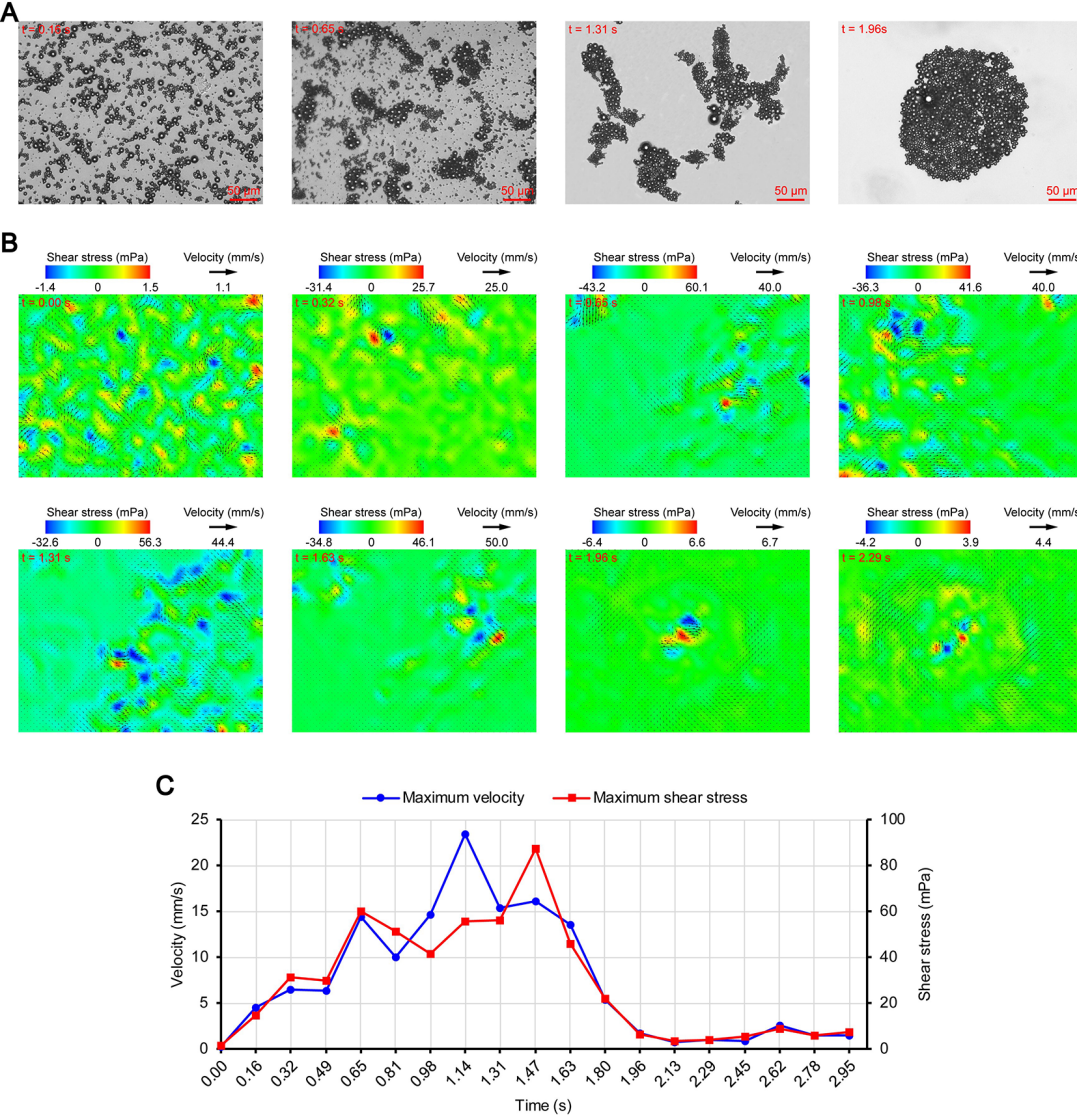
Micro-PIV results of the 0.35 MPa group. **(A)** Acoustic behavior of the microbubbles. **(B)** Temporal evolution and spatial distribution of the velocity fields and shear stress. **(C)** Time variation of the maximum shear stress and maximum flow velocity.

**Figure 5:**
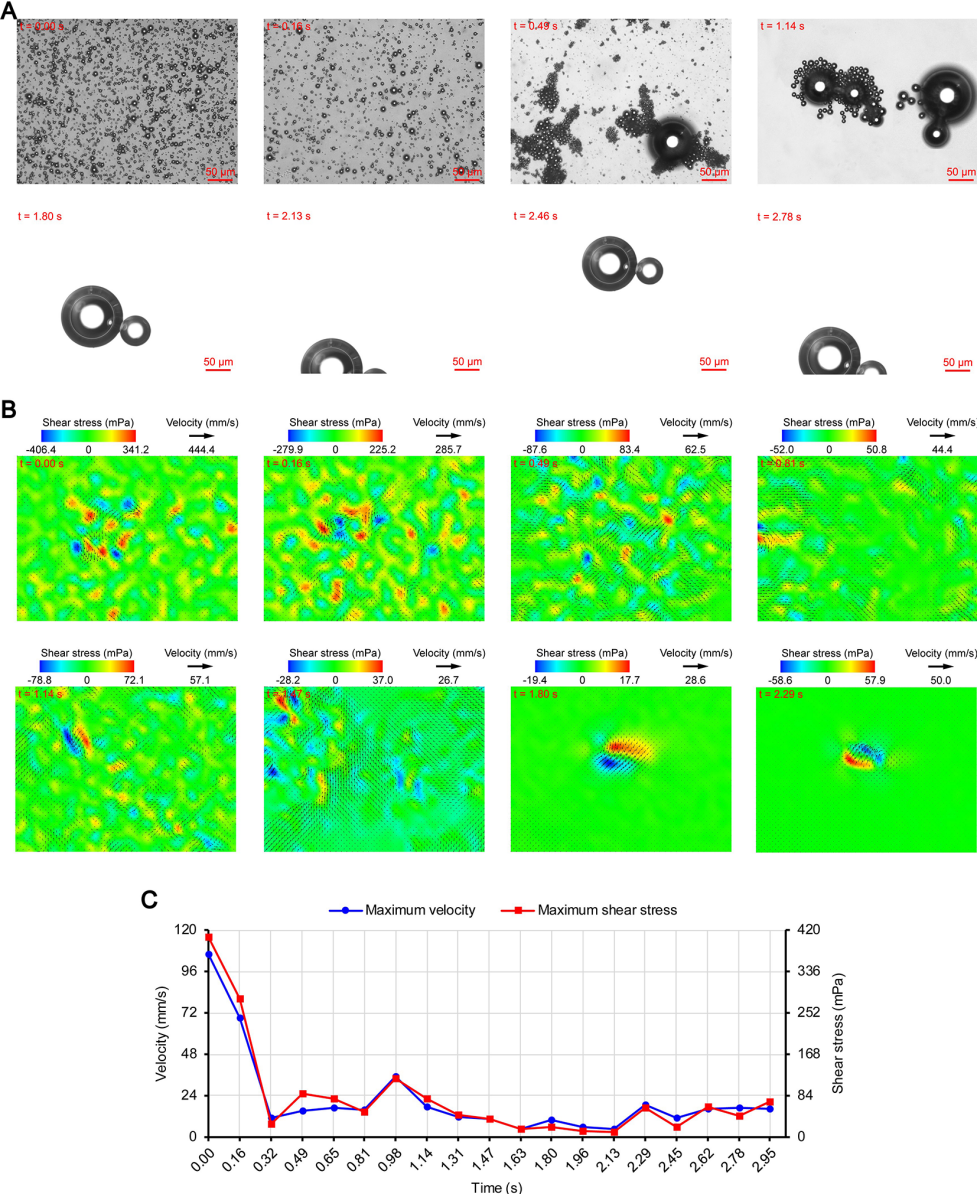
Micro-PIV results of the 0.70 MPa group. **(A)** Acoustic behavior of the microbubbles. **(B)** Temporal evolution and spatial distribution of the velocity fields and shear stress. **(C)** Time variation of the maximum shear stress and maximum flow velocity.

**Figures 3A**, **4A**, and **5A** show the acoustic behavior of the microbubbles of the three experimental groups in the ultrasound field. Once ultrasound was applied, the dispersed microbubbles in the 0.20 and 0.35 MPa groups rapidly aggregated into small clusters. The small clusters then gathered together to form several honeycombed microbubble clusters of different sizes at 3.93 and 1.96 s, respectively. Some researchers have referred to this kind of microbubble cluster as a microbubble cloud (Kotopoulis and Postema, 2010). The resulting microbubble clouds were relatively static and showed no obvious movement. For the 0.70 MPa group, it was found that the concentration of microbubbles in the second image frame (0.16 s in **Figure 5A**) is approximately 32.2% lower than that in the first image frame (0.00 s in **Figure 5A**) by evaluating the changes in the number of pixels occupied by the microbubbles. This indicates that the microbubbles were partially destroyed. Subsequently, the remaining microbubbles underwent clustering and coalescence. The clustering refers to the deduction of distance among microbubbles, while the coalescence means the fusion of two or more microbubbles. Finally, the microbubbles formed several bubble clusters which underwent translating oscillation along a single axis (1.80–2.78 s in **Figure 5A**) (Tho et al., 2007).

The action of mechanical force caused the above microbubble behavior. In this experimental system, the microbubbles were mainly affected by five kinds of mechanical forces: the buoyancy, gravity, primary radiation force, secondary radiation force, and sound pressure. The primary radiation force acts on an object in a sound field parallel to the direction of the sound (Dayton et al., 1997; Dayton et al., 2002). Therefore, the microbubbles were subjected to the downward gravity and primary radiation force as well as the upward buoyancy. With ultrasound irradiation, no sinking microbubbles were obvious in the other planes of the fluid, which indicates that the buoyancy was greater than the sum of the gravity and primary radiation force. This caused the microbubbles to always float on the detection plane. Therefore, the behavior induced by these three mechanical forces was slight.

As described in the introduction, sound pressure with positive and negative variations causes cavitation of microbubbles. Although the details of the microbubble oscillations could not be observed because of the low magnification of the objective lens, the type of cavitation could be distinguished depending on whether or not the microbubbles were broken. Therefore, the 0.20 and 0.35 MPa groups underwent stable cavitation, while the 0.70 MPa group underwent transient cavitation in the initial stage of ultrasound irradiation. This was consistent with the report by Lin et al. (2017) on the transient cavitation threshold of SonoVue microbubbles. We also noticed that the smaller microbubbles preferentially ruptured, probably due to their lower transient cavitation threshold (Kooiman et al., 2014). In addition to the cavitation, sound pressure also induces microbubble coalescence (**Figure 5A**). The expansion or collision of adjacent microbubbles can result in flattening and thinning of the phospholipid coatings at the contact (Postema et al., 2004). These deformations continue until a critical thickness of about 0.1 µm is reached, at which the van der Waals force results in coating rupture and microbubble coalescence (Kotopoulis and Postema, 2010). The degree of deformation is positively correlated with the PRP (Kotopoulis and Postema, 2010), so obvious coalescence was observed only in the 0.70 MPa group.

The secondary radiation force is also called the Bjerknes force and is generated by the scattering effect of the incoming ultrasonic waves from the liquid-gas interface (Hashmi et al., 2012), which causes the originally dispersed satellite bubbles to gather toward the core bubbles and form microbubble clusters (Fan et al., 2014). Kotopoulis and Postema (2010) demonstrated that the time required for clustering was inversely proportional to the square of the PRP. However, the clustering time ratio of the 0.35 MPa group to the 0.20 MPa group was 1.96/3.93 = 0.4987, which did not match the ratio of the square of the PRP (0.35^2^/0.20^2^ = 3.0625). The main reason may be that the boundary layer limited the acceleration of the microbubble clustering. Another possible reason is that the attraction of the microbubbles to the tracer particles reduces the measurement accuracy. Under the secondary radiation force, the particles are attracted toward the microbubbles (Hashmi et al., 2012), which may cause the measured value of the flow velocity to be lower than the true value. In order to avoid this influence, we chose particles that were smaller than those used by Cho et al. (2015). The PRP of the 0.70 MPa group was significantly higher than that of the 0.35 MPa group, but the clustering time of the two groups were very close. This may be because the concentration of the microbubbles decreased significantly after transient cavitation and the secondary radiation force decreased accordingly.

### Relationships Between the Acoustic Behavior of Microbubbles and Flow Fields

After the laser was turned on, the flow tracers of the detection plane emitted dot-like fluorescence, and no obvious aggregation was observed (**Figure 2C**). The instantaneous velocities of the detection plane were obtained through analysis of the fluorescence signal. The maximum velocities of the PBS and MB groups were 0.006 and 0.008 mm/s, respectively, and the maximum shear stress of both groups was 0.02 mPa (**Figures 2D, E**). The average uncertainty of the measured velocity is 5.6×10^−2^ mm/s, which is calculated by estimating an uncertainty in pixel displacement to be ±0.1 pixels (Raffel et al., 2007; Liu et al., 2019). Thus, the maximum velocities of the flow fields were less than the detection range of the micro-PIV when no ultrasound was applied, and the measured velocities should be regarded as background noise. For the US group, although no microbubbles were added, the flow fields changed significantly with a maximum velocity of 4.5 mm/s and maximum shear stress of 14.9 mPa after ultrasound was applied (**Figure 2F**). This may have been due to the cavitation of the naturally dissolved air in the fluid. In order to test the hypothesis, the suspension of the US group was degassed and subjected to ultrasound again. The flow fields recovered to the levels of the PBS and MB groups, which confirmed the above conjecture.

**Figures 3B**, **4B**, and **5B** show the temporal evolution and spatial distribution of the velocity fields and shear stress of the three experimental groups. Before the formation of microbubble or bubble clusters (0.00–3.93 s in **Figure 3B**, 0.00–1.96 s in **Figure 4B** and 0.00–1.80 s in **Figure 5B**), none of the three groups showed obvious flow pattern and shear stress distribution law. Experimental studies have shown that the flow fields generated by the dynamic behavior of a single bubble have a specific pattern (Tho et al., 2007; Collis et al., 2010; Reuter et al., 2017). Although the bubbles they studied differ from the SonoVue microbubbles in size and shell, Collis et al. (2010) argued that nonlinear phenomena such as microstreaming are similar. However, when the flow fields of multiple microbubbles are superimposed on each other, the respective patterns are masked, which results in an unsteady flow overall without a specific pattern.

After the clustering was completed, multiple microbubbles formed a whole body that performed a unified motion, and the flow fields showed a relatively clear pattern as follows. The fluids of the 0.20 and 0.35 MPa groups diverged around the microbubble cloud, and the fluid velocity and shear stress decreased with increasing distance from the microbubble clouds. For the 0.70 MPa group, the flow direction was consistent with the movement direction of the bubble clusters, and the fluid velocity decreased with increasing distance from the bubble clusters. The shear stress on both sides of the bubble cluster was symmetrically distributed with similar values and opposite directions (1.80 s in **Figure 5B**). When the movement direction was reversed, the flow direction was simultaneously reversed, and eddy currents formed on both sides of the bubble cluster. Meanwhile, the direction of the shear stress also reversed and was still symmetrically distributed (2.29 s in **Figure 5B**).

The relationship between UMDD and delivery has often been discussed in the literature, and the shear stress threshold for sonoporation is one of the most commonly studied parameters (Wu et al., 2002; Fan et al., 2014; Helfield et al., 2016b; Nejad et al., 2016; Rong et al., 2018). **Figures 3C**, **4C**, and **5C** show the time variation of the maximum shear stress. Although not absolute, a high shear stress tends to correspond to high flow velocity, so the time variation of the maximum flow velocity is also shown in these figures. In the early stage of the 0.20 and 0.35 MPa groups (0.00–2.13 s in **Figure 3C** and 0.00–0.65 s in **Figure 4C**), the maximum velocity and maximum shear stress gradually increased to a high level, and the acceleration of both increased with time. The main reason was that, as the microbubbles continued to gather, the secondary radiation force increased accordingly (Fan et al., 2014), which in turn produced greater acceleration. After a brief fluctuation at a high level, the maximum velocity and maximum shear stress decreased rapidly, which indicated that the momentum of each cluster was significantly offset in the final stage of clustering. Although microbubbles can undergo linear and nonlinear oscillation at a PRP that is lower than the transient cavitation threshold, no obvious movement of the microbubble clouds was observed, and the shear stress were also very weak (9.1 mPa and 8.7 mPa, respectively). Studies (Caskey et al., 2007; Garbin et al., 2007; Overvelde et al., 2011) have shown that, when a bubble is near a wall, its cavitation is significantly suppressed. Therefore, the reason for the above phenomenon appears to be that the stacking and squeezing of microbubbles inhibited the oscillation.

Once ultrasound was applied, the maximum velocity and maximum shear stress of the 0.70 MPa group were immediately increased to 105.8 mm/s and 406.4 mPa, respectively. The sound pressure and secondary radiation force act on microbubbles at the same time, but the time scales of the two actions are completely different. After ultrasound was turned on, ultrahigh-speed microscopy was used to observe the occurrence of transient cavitation within tens of microseconds (Prentice et al., 2005), while the clustering take several seconds (Kotopoulis and Postema, 2010). Therefore, in the initial stage of ultrasound irradiation, the microjets generated by transient cavitation were the main components of the flow fields. When the microjets were perpendicular to the detection plane (Brujan et al., 2001), their accompanying eddy currents could be detected by micro-PIV (Reuter et al., 2017). Although the eddy currents were much weaker than the microjets which ranged in speed from a few meters per second (Prentice et al., 2005; Reuter et al., 2017) to hundreds of meters per second (Brujan et al., 2001), the former still had the highest flow velocity and shear stress measured in this study. The duration of the eddy currents was short, so the maximum velocity and maximum shear stress rapidly decreased to a low level (0.00–0.32 s in **Figure 5C**). Thereafter, the maximum velocity and maximum shear stress gradually increased to a high level and then rapidly decreased, and fluctuated between 9.9–71.7 mPa (0.32–2.95 s in **Figure 5C**).

The acoustic behavior of the microbubble group is very complicated because of the influence of the incident ultrasound, acoustic scattering, acoustic radiation force, and other factors (Lin et al., 2017); thus, flow field data measured under the same conditions may vary greatly. To account for this, the present study focused on the change law of the flow velocity and shear stress rather than the magnitude of specific values. The flow field detection of each group was repeated three times, and the change law was basically the same.

### Relationship Between the Flow Fields and Delivery

FITC was used as a fluorescence marker for identifying sonoporated cells (Lin et al., 2018) in this study because it normally would not permeate through the cell membrane unless the membrane permeability is increased by sonoporation. PI, a cell viability detected agent, was used to distinguish the type of sonoporation (Lin et al., 2018) because the two types of sonoporation had different effects on cell viability. In conclusion, both FITC and PI positive indicates the occurrence of irreversible sonoporation, while FITC positive and PI negative represent reversible sonoporation.

**Figure 6:**
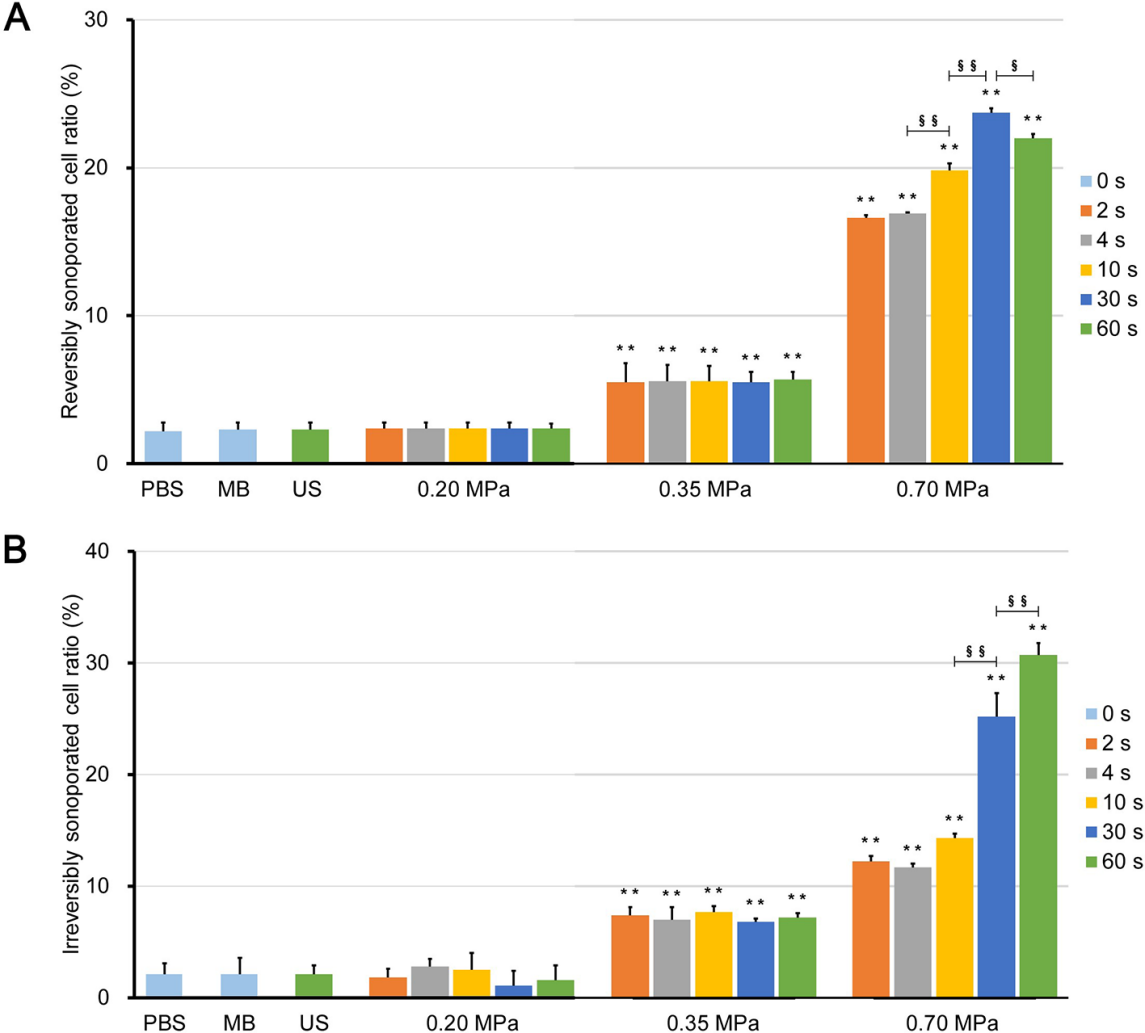
Ratio of **(A)** reversibly sonoporated and **(B)** irreversibly sonoporated cells. Data are shown as the mean ± SEM (n = 3). ** *p* < 0.01 vs the control and 0.20 MPa group; ^§^*p* < 0.05, ^§§^*p* < 0.01.

**Figures 6A, B** show the rates of reversibly and irreversibly sonoporated cells, respectively. The two rates of the three control groups were very low, which indicates that no significant FITC uptake and cell death occurred. Although the flow fields of the US group were completely different from those of the PBS and MB groups, there was no statistical difference between the three control groups (*p* > 0.05), which indicated that ultrasound combined with naturally dissolved air cannot cause obvious biological effects.

There was also no significant difference between the 0.20 MPa group and control groups (*p* > 0.05), which indicates that the maximum shear stress (**Figure 3C**) was less than the shear stress threshold for sonoporation. Although both the magnitude of the shear stress and the exposure time are important factors for sonoporation, the experimental results showed that prolonging the ultrasound exposure time to 60 s did not change the FITC uptake efficiency and cell viability significantly (*p* > 0.05) because of the weak shear stress generated by microbubble clouds.

After ultrasound irradiation for 2 s, the rates of the reversibly and irreversibly sonoporated cells of the 0.35 MPa group increased to 5.5% ± 1.3% and 7.4% ± 0.7%, respectively. This suggests that the maximum shear stress of the 0.35 MPa group exceeded the shear stress threshold for sonoporation in the accumulation stage, and both types of sonoporation occurred. The magnitude of the shear stress generated by microbubbles driven by low acoustic pressure and the shear stress threshold for sonoporation vary widely with a range from millipascals to kilopascals (Wu et al., 2002; Fan et al., 2014; Helfield et al., 2016b; Nejad et al., 2016; Rong et al., 2018). The primary reason for this difference is that the researchers used different calculation models and the current experimental or theoretical methods cannot account for all stress components (Ma et al., 2015). In the present study, the wall shear rate was estimated from the near-wall velocity, which may have affected the measurement of the wall shear stress (Cho et al., 2015). Despite this, the variation in the shear stress was credible. In addition, prolonging the ultrasound exposure time also failed to significantly change the flow cytometry results of the 0.35 MPa group for the same reason as the 0.20 MPa group.

The 0.70 MPa group underwent transient cavitation, and microjets directed toward cells were formed when microbubbles collapsed. The shear stress of the microjets was on the order of megapascals (Liang et al., 2010; Kooiman et al., 2014), which far exceeded the sonoporation shear stress threshold. Therefore, after ultrasound irradiation for 2 s, the rates of reversibly and irreversibly sonoporated cells further increased to 16.6% ± 0.2% and 12.2% ± 0.5%, respectively. It should be noted that there are differences between *in vitro* cell experiments and practical applications *in vivo*. Typically, the elastic modulus of vessels is below 1 MPa, which indicates that the microjets are more likely to point away from the vessel wall (Brujan et al., 2001). This has been experimentally verified in *ex vivo* rat mesentery (Chen et al., 2011). Borosilicate glass with an elastic modulus of 7.2 × 10^4^ MPa was used in the present study which caused the microjets to point towards the glass. When the exposure time to 30 s, both rates continued to increase to 23.7% ± 0.3% and 25.2% ± 2.1%, respectively; this indicated that the flow fields generated by the finally formed bubble clusters could induce sonoporation. When the exposure time was further increase to 60 s, the rate of irreversibly sonoporated cells increased to 30.7% ± 1.1%, while the rate of reversibly sonoporated cells decreased to 22.0% ± 0.3%. The reason may be that, under the action of shear stress, reversible sonoporation occurs first; as the action time increases, the reversible pores turn into irreversible ones, which leads to cell death. This is consistent with the point proposed by Wu et al. (Wu et al., 2002) that the mechanisms of reversible and irreversible sonoporation may be similar, but the degree of damage to cell membranes is different. A major challenge to the application of UMDD is both to obtain high delivery and to maintain good cell viability (Mehier-Humbert et al., 2005), and the above findings may be helpful in resolving this issue.

## Conclusion

UMDD is a complex process and research on its mechanism requires cooperation between multiple disciplines. In this study, flow visualization technology was used to explore the change law of the flow fields generated by SonoVue microbubbles and the relationships among the ultrasound parameters, acoustic behavior of microbubbles, flow fields, and delivery results. Results indicate that under different ultrasonic conditions, SonoVue microbubbles exhibit different acoustic behavior that generate various flow fields which lead to distinct delivery results. These findings effectively serve to substantiate the causal relationship between flow fields and sonoporation and contribute to the clinical application of UMDD.

One limitation of this study is that only the PRP was selected as the variable. Other ultrasound parameters may also have an effect on the sonoporation. For example, when excited by ultrasound at resonance frequency, a bubble will result in the maximum acoustic radiation forces and the maximum shear stress (Kooiman et al., 2014). However, there is not one single resonance frequency for a polydisperse population of microbubbles. This means that only a subset of microbubbles will resonate and the oscillation amplitude of other microbubbles is much lower. Moreover, this study did not delve into the temporal and spatial relationships between the flow fields and cellular response, which will be the main objective of future research.

## Data Availability Statement

The datasets generated for this study are available on request to the corresponding author.

## Author Contributions

PZ and ML conducted the micro-PIV experiments. PZ drafted the manuscript. PZ, ZW, and GZ carried out the UMDD delivery experiments. YP, LD, YD, ZL, and QS contributed to conception and design of the study. All authors contributed to manuscript revision and approved the submitted version.

## Funding

This work was supported by the National Natural Science Foundation of China (No. 81571677) and the Three-year Plan for Clinical Skills and Innovation in Municipal Hospitals (No. 16CR3093B).

## Conflict of Interest

The authors declare that the research was conducted in the absence of any commercial or financial relationships that could be construed as a potential conflict of interest.
