# Supplementary material for: Micro-Particle Image Velocimetry Investigation of Flow Fields of SonoVue Microbubbles Mediated by Ultrasound and Their Relationship With Delivery
Source: Front Pharmacol. 2020 Jan 28;10:1651. doi: 10.3389/fphar.2019.01651 (PMC7025580; doi:10.3389/fphar.2019.01651)
Supplement: Supplementary file 1 [file DataSheet_1.docx]

Supplementary Material

# Details of the acoustic calibration

The tank was filled with degassed water, and a hydrophone was placed on the upper surface of the cell culture chamber (Figure S1A). The 2D profile of the PRP was mapped by moving the hydrophone with a three-axis translation stage (AST3-S, ONDA, USA). The measured spatial PRP for an output intensity of 2.00 W/cm^2^ is shown in Figure S1B. It can be seen that the spatial distribution of PRP on the upper surface of the culture chamber is relatively uniform at about 0.35 MPa. Three independent measurements were performed to yield the output intensity-PRP curve shown in Figure S1C. A positive correlation can be observed between the PRP and output intensity. It should be noted that because of the volume limitation, the hydrophone was not inserted into the chamber. According to our research, the 0.13 mm thick glass plate was too thin to cause significant acoustic attenuation.

## Supplementary Figures


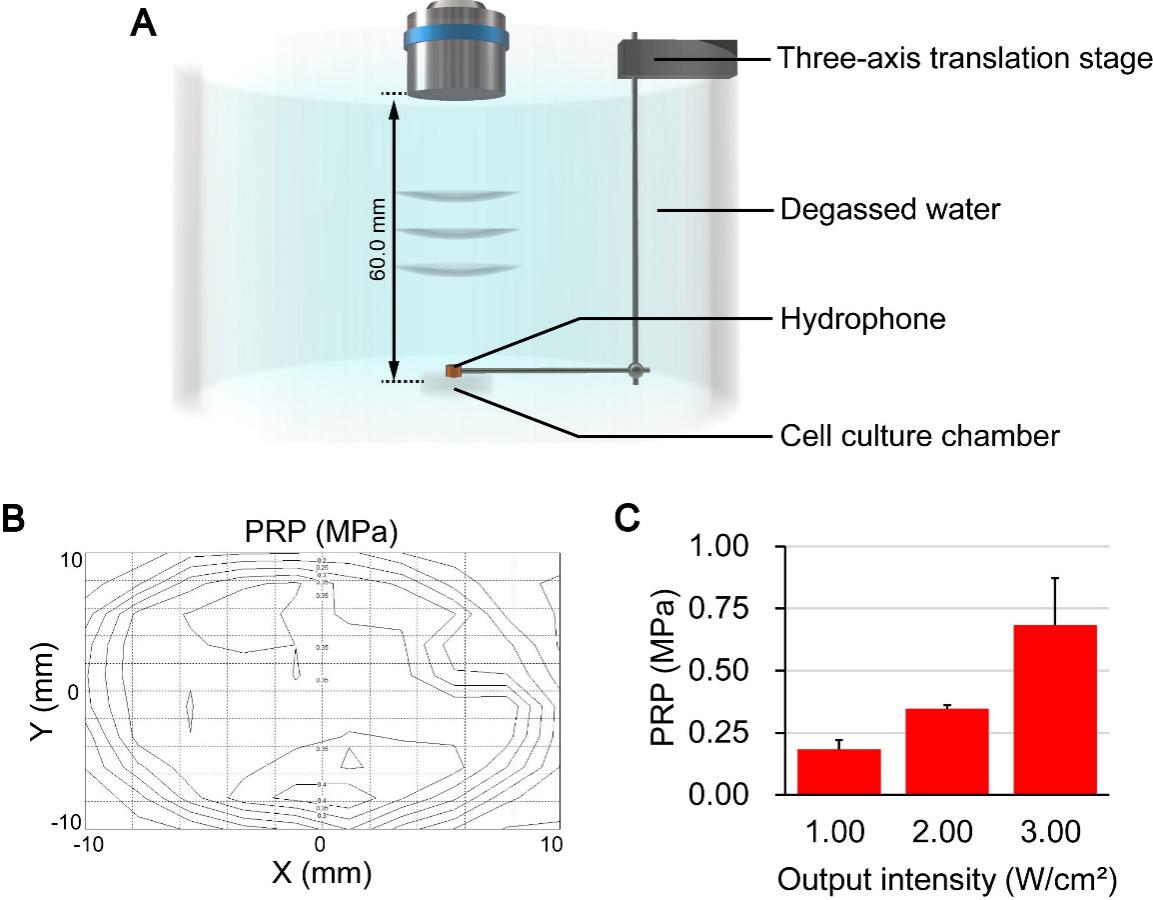


**Figure S1** Schematic representation of acoustic calibration: **(A)** experimental apparatus for PRP detection; **(B)** 2D profile of the PRP on the upper surface of the culture chamber; **(C)** PRP as a function of transducer output intensity. The other ultrasound parameters were fixed, including a center frequency of 1 MHz, pulse repetition frequency of 100 Hz, and duty cycle of 1:5.
